# Metastatic melanoma to the testis

**DOI:** 10.1259/bjrcr.20170104

**Published:** 2018-02-22

**Authors:** Madhavi Patnana, Brinda Rao Korivi, Catherine E Devine, Silvana Faria, Victor Prieto, Merrick I. Ross, Chaan S. Ng

**Affiliations:** 1 Department of Diagnostic Radiology, Anatomical, University of Texas MD Anderson Cancer Center, Houston, TX, USA; 2 Department of Pathology, Anatomical, University of Texas MD Anderson Cancer Center, Houston, TX, USA; 3 Department of Surgical Oncology, University of Texas MD Anderson Cancer Center, Houston, TX, USA

## Abstract

This case report presents the ultrasound and positron emission tomography-computed tomography (PET-CT) imaging findings related to a patient with metastatic melanoma to the testis. We review this very rare entity and discuss the role of imaging.

Differential considerations for a patient presenting with scrotal pain and swelling are quite wide, ranging from benign to malignant processes. The former include such entities as testicular torsion and epididymo-orchitis, and the latter, primary and secondary malignancies.^1^ Distinction between these entities is critically important because treatment pathways vary widely according to the precise diagnoses.

Metastatic melanoma to the testicle has only rarely been reported,^2–4^ and there have been very limited previous descriptions of its imaging findings.^5, 6^ To the best of our knowledge, there have been no previous radiological reports describing both ultrasound and PET-CT imaging findings in the same patient.

## CLINICAL PRESENTATION

A 71-year-old male presented with increasing pain and swelling in the scrotum over several days and weeks. There was no history of trauma, dysuria or fever.

2 years prior to presentation he had a shave biopsy of a cutaneous lesion in his upper back, which was found to be a melanoma, at least 2.3 mm in thickness. Owing to the lack of an obvious intraepidermal component, the diagnosis of melanoma included a comment about the possibility of that lesion being a cutaneous metastasis from an unknown origin. He subsequently underwent a wide local excision and sentinel lymph node mapping and biopsy; the latter was negative for metastatic disease. 3 months after the initial excision, investigations revealed subcutaneous metastatic disease to the right flank and lower back, and the patient was started on systemic chemotherapy. The treatment consisted of temozolomide, which was subsequently combined with sorafenib, for a total of 6 months. He then underwent excision of the subcutaneous metastases in order to harvest tissue in preparation for a clinical trial of bioimmunotherapy. However, 3 months following the excision, he developed new subcutaneous nodules, pulmonary and axillary nodules, and small bowel lesions. The latter were found to be metastatic implants at the time of a palliative small bowel resection. Following the bowel resection, he developed a wound infection, urinary tract infection and pneumonia, for which he received various antibiotics based on culture sensitivities. The patient had a remote history of a right orchidectomy, but he was unable to provide details as to the reason for this procedure.

On the current presentation, the patient reported that he had scrotal pain at the time of his small bowel resection, 1 month previously, and that it had subsided briefly; however, the pain and swelling had now recurred and were intensifying. Physical examination revealed an inflamed scrotum and a tender 5–6 cm non-fluctuant mass in the left testicle.

## DIFFERENTIAL DIAGNOSIS

Differential considerations in general for a testicular mass include tumours, inflammatory processes (*e.g.* epididymo-orchitis) and testicular torsion. There is overlap of imaging appearances of testicular tumours and non-cancerous processes (*e.g.* infarction, haematoma and infection/abscess), which can appear as mass-like hypoechoic areas with variability of internal blood flow.^1^

Testicular tumours may be primary or secondary. Primary testicular cancers, which may be germ cell or non-germ cell, are the most common tumours in males aged 15–34 years and account for 1% of all cancers in males.^7^ Germ cell tumours are categorized into two major groups, seminomatous or non-seminomatous, accounting for 90–95% of primary testicular tumours.^1^ Sex cord stromal tumours comprise the majority of non-germ cell tumours and include Leydig cell and Sertoli cell tumours and other rare subtypes such as granulosa cell tumours and gonadoblastoma.^1^

In males over 60 years of age, tumours to be considered include lymphoma, metastases and other rare tumours, the most common being non-Hodgkin’s lymphoma. The presence of bilateral disease favours malignant processes, such as lymphoma, leukaemia or metastases. Lymphoma is also the most common bilateral testicular tumour.^8, 9^ Leukaemia and lymphoma have similar ultrasound imaging appearances, including a homogeneously hypoechoic echotexture or multiple varying sized hypoechoic foci throughout the testicular parenchyma. These patients often present with an enlarged testis.^1^

Metastatic disease to the testicle is uncommon; however, it is a primary consideration in a patient of this age and melanoma history. The most common primary tumour to metastasize to the testes is from the prostate (35%), followed by lung (19%), melanoma (9%), colon (9%) and kidney (7%), as described by Richie et al.^4^ Metastatic melanoma to the testis is usually found at autopsy. A retrospective study of 738 autopsies of adult males with known solid malignant neoplasms revealed a prevalence of metastatic disease to the testes of only 0.68% (5/738).^3^ In their study, three of the five positive cases were from lung, and one each from pancreatic islet cell tumour and melanoma.

Rare tumours to the testicle include sarcoma, fibroma, neurofibroma, leiomyoma, vascular tumours and leukaemia.^10–12^

## IMAGING FINDINGS

**Figure 1.**
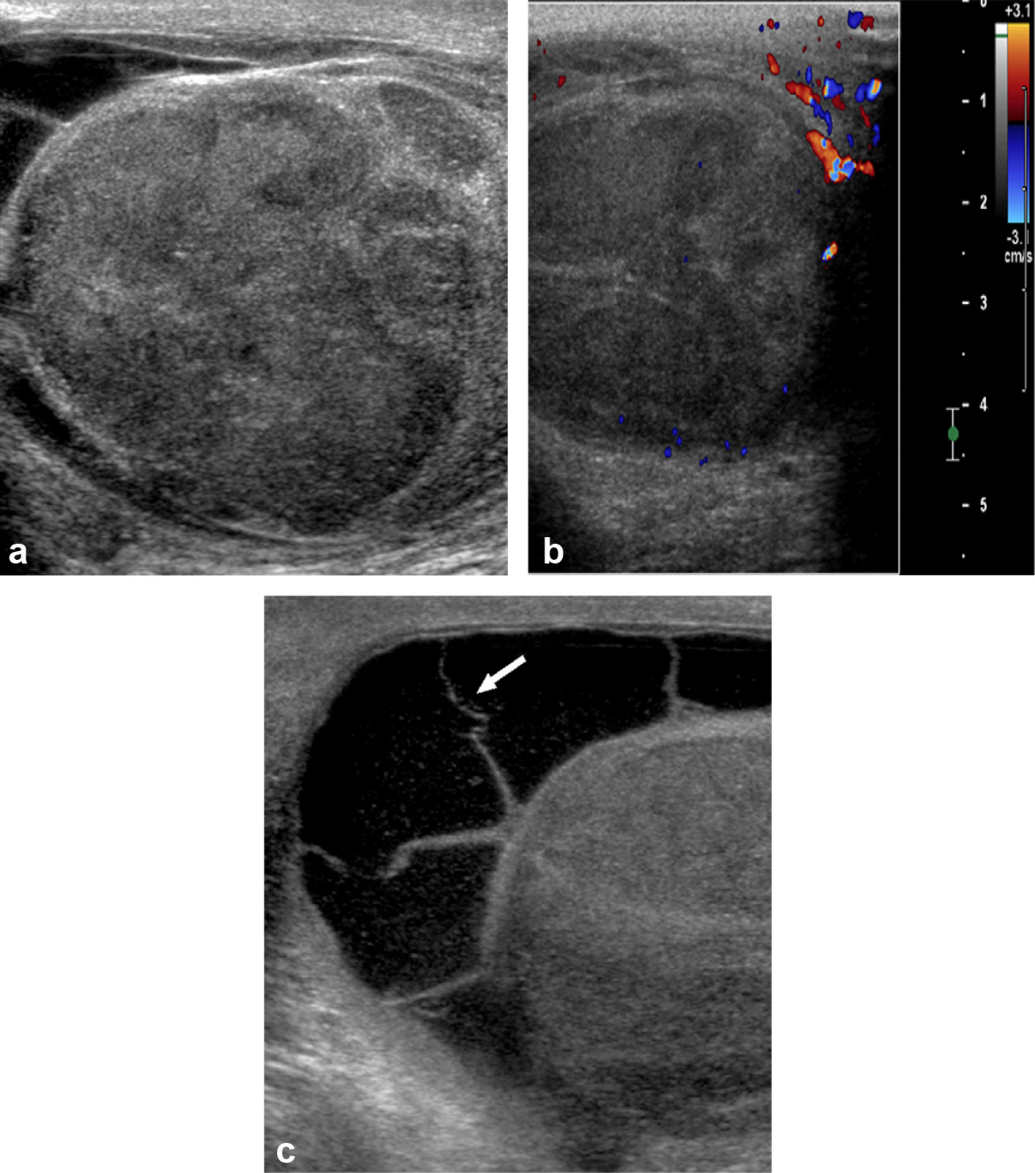
71-year-old male with scrotal pain and swelling. (a) Longitudinal sonographic image of the left testicle demonstrates a heterogeneous lobulated mass replacing the testicle. (b) Longitudinal sonographic image of the left testicle with colour Doppler demonstrates peripheral vascularity of the testicular mass. (c) Transverse sonographic image of the left testicle demonstrates a complex loculated hydrocele with septations (white arrow) and echogenic debris.

**Figure 2.**
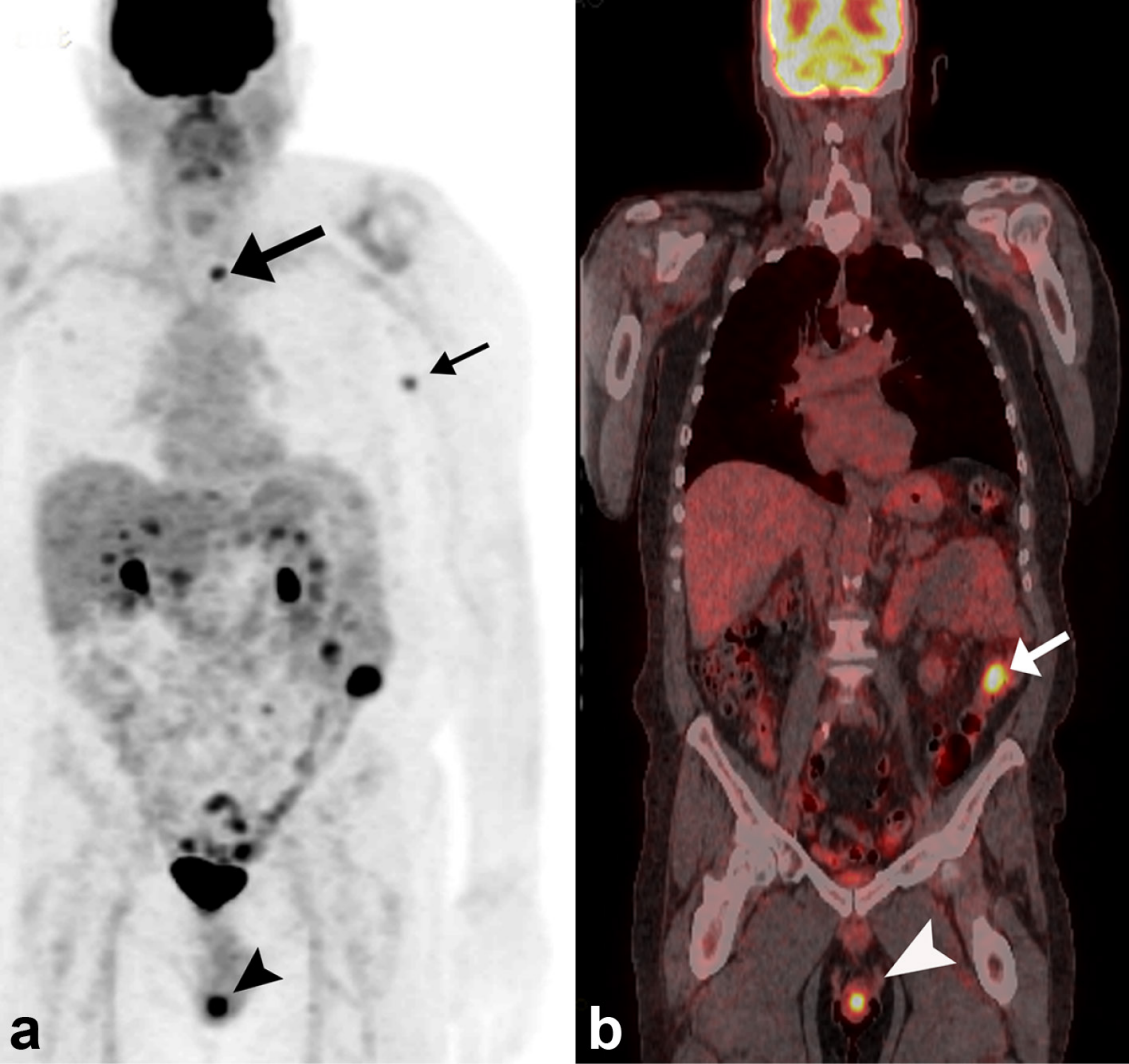
Positron emission tomographyPET-CT examination 8 months prior to presentation with scrotal symptoms. (a, b) Maximum intensity projection and fused coronal images demonstrate focal fludeoxyglucose uptake within the scrotum, corresponding to the left testicle, with an SUV_max_ of 12 (black and white arrow heads). Focal uptake is also noted in the left axilla from a metastatic subcutaneous nodule (small black arrow) and in the left transverse process of T4 vertebral body (large black arrow). (Focal uptake in the descending colon (white arrow) was not associated with any abnormality on subsequent colonoscopy).

An ultrasound of the scrotum demonstrated an enlarged left testicle with a 5 cm lobulated, heterogeneous, solid mass replacing the testicle with some peripheral vascularity on colour Doppler (Figure 1). A loculated left hydrocele with echogenic debris was also noted (Figure 1c). Retrospective review of a PET-CT staging examination, performed 8 months prior to scrotal symptoms, showed focal fludeoxyglucose (FDG) uptake within the scrotum (Figure 2) with a maximum standardized uptake value (SUV) of 12.

**Figure 3.**
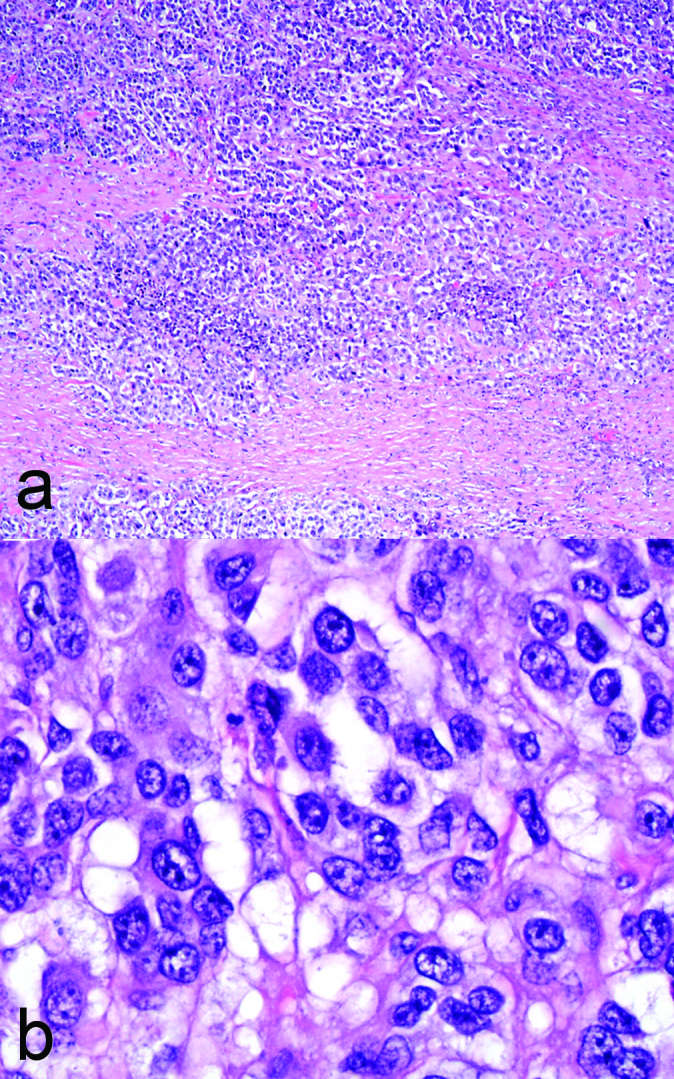
Histopathology sections of the testicular lesion. (a) Low-power image showing tumour cells almost completely obliterating the testicular parenchyma. (b) High-power image showing cells with large, epithelioid cytoplasm, and large nuclei and focal prominent nucleoli. (Haematoxylin and eosin, x4 and x40, original magnification).

Even though this patient had only one testicle, a palliative left orchidectomy, nonetheless, was undertaken for relief of symptoms. Evaluation of the gross specimen revealed that nearly the entire testicular parenchyma was replaced by an expansile, friable, focally rubbery grey to dark pink haemorrhagic 7.5 cm nodule, with only scant amount of normal testicular tissue. Histopathological evaluation demonstrated poorly cohesive, large, epithelioid cells with prominent nucleoli (Figure 3) involving the interstitium and focally the seminiferous tubules. There was also prominent vascular invasion by the tumour cells. There was no tumour in the epididymis or spermatic cord. A diagnosis of metastatic melanoma was established on histopathological examination after multiple immunohistochemical analyses. Interestingly, the metastasis to the testis, although morphologically similar to the other sites of metastasis (*e.g.* axilla and bowel), had lost most immunohistochemical expression of most melanocytic markers (MART1, HMB45 antigen, MITF).

## DISCUSSION

Melanoma is a neoplasm that arises from melanocytes and is increasing in incidence. In the United Kingdom, there were 15,400 new cases in 2014, and 2500 deaths from melanoma.^13^ It is estimated that in the United States in 2018, a total of 91,270 new cases of melanoma will be diagnosed (55,150 in males and 36,120 in females) and 9320 patients are expected to die of melanoma.^14^ The rise in incidence is thought to be two-fold: early detection through screening and increased exposure to ultraviolet light.^15^

Most melanomas arise from the skin; however, they can also develop in other locations such as the eye and mucosae.^16, 17^ Primary melanoma arising from the visceral organs is extremely rare, especially from the testicle.^4^ To the best of our knowledge, there has only been one reported case of primary testicular melanoma.^18^ Metastatic melanoma to the testicle is also extremely rare.^2, 4,6^ In a patient of this age with history of metastatic melanoma, although rare, a testicular melanoma metastasis should be a primary consideration.

Sonography is the imaging modality of choice for evaluating acute and non-acute scrotal disease. Ultrasound can evaluate intratesticular versus extratesticular lesions, as well as assess testicular vascularity, and has the capability to evaluate between solid or cystic lesions.^19, 20^ Comparison with the contralateral testicle can often be useful in evaluating the echogenicity. However, comparison with the contralateral testicle could not be performed in this case owing to prior history of surgical resection of the right testis. In our case, ultrasound revealed a heterogeneous mass, as described in another report of metastatic melanoma to the testicle.^6^

Melanomas are typically hypermetabolic, which contributes to the utility of PET-CT in the staging evaluation of metastatic disease.^21^ Retrospective review of a staging PET-CT examination performed 8 months prior to our patient’s presentation with scrotal pain revealed focal scrotal hypermetabolism above physiological activity, initially not reported. At that time, the patient had other reported FDG-avid subcutaneous and osseous metastases and did not have testicular symptoms. The difficulty for interpreters of PET-CT evaluations is that some degree of physiological metabolic activity can be detected in the testis of most males. A study of 86 testes in 70- to 79-year-old males has shown a mean maximum SUV (SUV_max_) of 2.18 ± 0.45 (range, 1.42–3.29).^22^ The SUV_max_ of 12 in our patient was substantially higher, and was similar to the SUV_max_ of 12.6 observed in another reported case of metastatic melanoma to the testicle.^5^ Hypermetabolic activity, however, cannot differentiate between inflammatory and neoplastic processes. FDG avidity can be seen in primary testicular neoplasms, testicular lymphoma and testicular metastases.^23, 24^

MRI of the scrotum has utility as a problem-solving tool in scrotal mass characterization, particularly in cases of non-diagnostic/inconclusive ultrasound examinations.^25^ If sufficient melanin is present, it can present as a high signal intensity mass on *T*_1_ weighted MRI images, an uncommon finding in other cancer types. Increased *T*_1_ signal could also be caused by haemorrhage.

Although sites of metastatic melanoma can be extensive and are frequently unpredictable, cutaneous melanoma usually metastasizes first to regional lymph node draining basins, before disseminating haematogenously. Our patient demonstrated signs of metastatic disease but absence of lymphatic/nodal disease during the entire course of his disease, which is unusual in primary cutaneous melanomas. Although the possibility of a metastasis from the testis to the skin was considered, this diagnosis was discarded based upon the interval between the clinical presentation of the lesion in the skin and the diagnosis of testicular involvement (~2.5 years).

In conclusion, this case report describes the imaging appearances of metastatic melanoma to the testis, and overall the imaging appearances are non-specific and essentially indistinguishable, based on both ultrasound and PET-CT, from other more common neoplastic testicular lesions, with the history in this case suggesting the diagnosis of metastatic melanoma.

Metastatic melanoma has the propensity to metastasize haematogenously to virtually any organ; some sites are more common than others, and melanoma rarely metastasizes to the testicle, with most cases found at autopsy. There have been very few reports describing the imaging findings of metastatic melanoma to the testicle, and in particular, to the best of our knowledge, there have been no previous reports describing the ultrasound and PET-CT findings of this entity in the same patient. Ultrasound may be helpful in refining the differential diagnosis in such situations, but is not specific. Although extremely rare, metastatic melanoma to the testis should be a consideration in a patient with scrotal pain and swelling, who has a history of malignant melanoma and presents with the imaging findings described in this report.

## LEARNING POINTS

1. Melanoma rarely metastasizes to the testicle, with most cases found at autopsy.
2. Focal hypermetabolism in the testicle greater than physiological activity on PET-CT should warrant further investigation.
3. Ultrasound is the modality of choice for evaluating acute and non-acute scrotal disease.
